# The Electron–Phonon Interaction at Vicinal Metal Surfaces Measured with Helium Atom Scattering

**DOI:** 10.3390/nano13232997

**Published:** 2023-11-22

**Authors:** Giorgio Benedek, Salvador Miret-Artés, Joseph R. Manson, Jan Peter Toennies

**Affiliations:** 1 Dipartimento di Scienza dei Materiali, Università di Milano-Bicocca, Via R. Cozzi 55, 20125 Milano, Italy; 2 Donostia International Physics Center (DIPC), Paseo de Lardizabal 4, 20018 Donostia-San Sebastián, Spain; s.miret@iff.csic.es (S.M.-A.); jmanson@clemson.edu (J.R.M.); 3 Instituto de Física Fundamental, Consejo Superior de Investigaciones Científicas, Serrano 123, 28006 Madrid, Spain; 4 Department of Physics and Astronomy, Clemson University, Clemson, SC 29634, USA; 5 Max-Planck-Institut für Dynamik und Selbstorganisation, Am Fassberg 17, D-37077 Göttingen, Germany; jtoenni@gwdg.de

**Keywords:** electron-phonon coupling, vicinal surfaces, helium atom scattering

## Abstract

Recently, it was demonstrated that inelastic helium atom scattering from conducting surfaces provides a direct measurement of the surface electron–phonon coupling constant (mass enhancement factor *λ*) via the temperature or the incident wave vector dependence of the Debye–Waller exponent. Here, previous published as well as unpublished helium atom scattering diffraction data from the vicinal surfaces of copper (Cu(11α), with α = 3, 5, 7) and aluminum (Al(221) and Al(332)) were analyzed to determine *λ*. The results suggested an enhancement with respect to the corresponding data for the low-index surfaces (111) and (001) above the roughening transition temperature. The specific role of steps compared to that of terraces is briefly discussed.

## 1. Introduction

Vicinal crystal surfaces, whose planes form a small angle with a low-index plane (e.g., (001), (111) or (110) in cubic crystals) have raised interest well beyond the pure crystallography field. Ideal vicinal surfaces are characterized by sets of equally spaced steps separated by low-index terraces. Their structure and stability with respect to faceting, reconstruction and roughening transitions have been the subject of several experimental studies with He atom scattering (HAS) and related theoretical investigations [1,2,3,4,5,6,7,8,9,10,11,12,13,14,15,16,17,18,19,20,21]. The early interest in vicinal surfaces was motivated by their expected role as natural templates for the epitaxial growth of functional nanostructures [22,23,24,25] and as heterogeneous catalysts [26]. The topological and the quasi-one dimensionality (quasi-1D) features of the steps are reflected in their electronic and vibrational properties, hence, in the local electron–phonon (el–ph) interaction, thus opening new horizons for quasi-1D superconductivity in topological materials [27,28,29,30].

In a previous work [31], we showed that the temperature or incident wave vector dependence of the Debye–Waller (DW) exponent in helium atom scattering (HAS) provides direct information on the total mass enhancement factor *λ*, characterizing the el–ph interaction at a conducting surface. The value of *λ* derived in this way (hereafter denoted as *λ_HAS_*) was reported for several metal surfaces [32], overlayers [33], graphene [34], topological semimetals [35,36,37,38], layered dichalcogenides [39,40], 2D superconductors [41] and low- and multi-dimensional surfaces [42].

**Figure 1:**
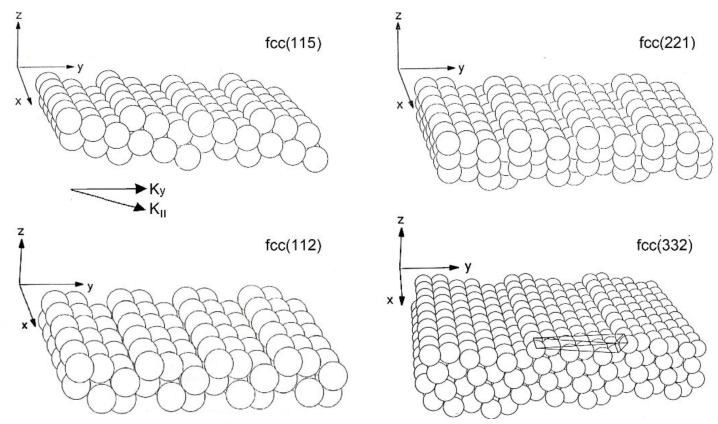
Ball models showing the structure of the (115) and (112) (**left**) and of the (221) and (332) (**right**) vicinal surfaces of a monatomic face-centered cubic (fcc) crystal (adapted from [47]). The orthogonal coordinates in the diagram are those of the vicinal surface; the wave vector component normal to steps lying in the terrace plane is here denoted as *K*_||_.

In this work, the study was extended to high-index metal surfaces, by re-analyzing previous HAS diffraction measurements on the vicinal surfaces of copper Cu(11α) (with α =1, 3, 5, 7 [43,44] and α = 2, 5 [45,46,47]) and aluminum Al(221) and Al(332) [47,48]. In Figure 1, ball models of the (115) and (112) (left side) and of the (221) and (332) (right side) vicinal surfaces are shown. The analysis revealed that the DW exponent −2*W*(*T,k_i_*), as a function of both the surface temperature *T* and the incident He beam wave vector *k_i_*, contained distinct information on the el–ph interaction associated with either steps or terraces. Previously, Lapujoulade et al. [43,44] observed that, with increasing temperature, the typical linear slope of 2*W*(*T,k_i_*) for Cu(11α) became suddenly steeper above a certain temperature *T_R_* of the order of or above room temperature, indicative of a surface roughening transition. It is shown in Section 3 that this kind of roughening transition actually yielded an increase in the local el–ph interaction, similarly to what was recently reported for a semiconductor surface [49].

## 2. Theory

The specular HAS intensity is written as a function of the incident wave vector *k_i_* and the surface temperature *T* in the form

(1) $$\begin{array}{l} I(k_{i},T)=I_{0}(k_{i})\;e^{-2W(k_{i},T)}\\ \;\;\;\;\;\;=I(k_{i},0)\;e^{-2W(k_{i},T)+2W(k_{i},0)} \end{array}$$

In the second line of this equation, the specular scattering intensity is more explicitly factorized into its value at *T* = 0 K times the exponential temperature-dependent attenuation factor, which is just 1 at 0 K. Note that the incident wave vector dependence of the pre-factors includes that of the incident intensity. This is conveniently described for small *k_i_* by a power law, $I_{0}(k_{i})\propto k_{i}^{\eta }$, where *η* depends on the atom–surface interaction as well as on the supersonic beam source design and operating conditions [31].

The present el–ph theory of HAS from conducting surfaces [31,32] links the surface el–ph mass enhancement factor *λ*_HAS_ to the dependence of the DW exponent on the temperature or the incident momentum through the two equations

(2) $$\lambda_{HAS}=-\frac{\pi \phi }{2n_{s}a_{c}}\frac{\partial \ln I(k_{i},T)}{k_{iz}^{2}\partial (k_{B}T)}$$

and

(3) $$\lambda_{HAS}=-\frac{\pi \phi }{2n_{s}a_{c}}\frac{\partial \ln[k_{i}^{-\eta }I(k_{i},T)]}{(k_{B}T)\partial k_{iz}^{2}},$$

respectively. Here, *ϕ* is the surface work function, *n_s_* is the effective number of the surface atomic layers involved in the surface el–ph interaction, and *a_c_* is the surface unit cell area. For vicinal surfaces, *a_c_* is approximated by that of the terraces between two neighbor parallel steps. The power law for $I_{0}(k_{i})$ ensures that the measured $I(k_{i},T)$ at a given surface temperature *T* vanishes for both *k_i_* → 0 and *k_i_* → ∞. It will therefore have a maximum at *k_i_* = *k_i_*_,max_*,* where $\partial I(k_{i,\max},T)/\partial k_{i}=0.$ By combining this condition with Equations (1) and (3), the latter can be expressed as

(4) $$\lambda_{HAS}=\frac{\pi \phi }{4n_{s}a_{c}}\frac{\eta }{k_{B}T\;k_{iz,\max}^{2}}.$$

Note that, by inserting Equation (4) into (3), $k_{iz,\max}^{2}$ can be obtained from

(5) $$\frac{1}{k_{iz,\max}^{2}}=-\frac{2\partial \ln[k_{i}^{-\eta }I(k_{i},T)]}{\eta \;\partial k_{iz}^{2}},$$

and is therefore dependent on the surface temperature.

As discussed in Ref. [31], when the effects of the attractive surface potential depth *D* are not completely negligible in specular HAS, they can be approximately accounted for by replacing $k_{iz}^{2}$ in Equation (2) with ${\bar{k}}_{iz}^{2}=k_{iz}^{2}+2mD/\hbar^{2}$ (Beeby correction [50]), where *m* is the He atom mass, and the potential depth *D* can directly be obtained from the analysis of HAS bound-state resonances [51]. Classically, the Beeby correction is equivalent to assuming that the attractive potential of the He–surface interaction contributes to the speed with which the atom impacts the surface. When the *k_i_* dependence of the HAS intensity is used instead of its temperature dependence to derive *λ*_HAS_, Equation (4) has the advantage that the information on the electron–phonon interaction is within the factor $k_{iz,\max}^{2}$, to which the Beeby correction can be directly added.

## 3. The Copper Vicinal Surfaces Cu(11α)

**Figure 2:**
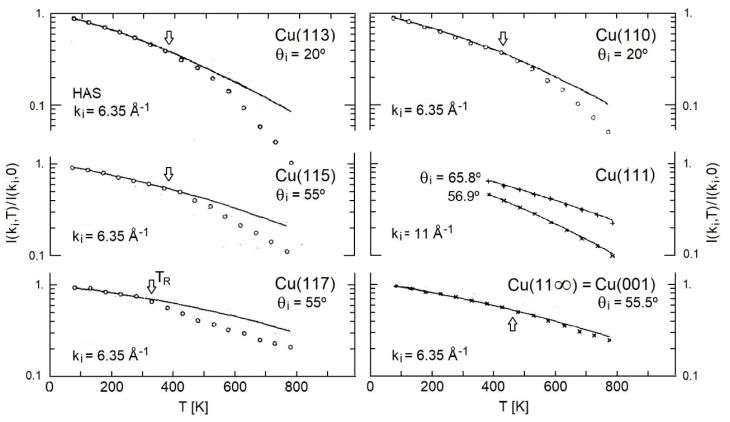
The specular HAS intensity as a function of temperature, normalized to the *T* = 0 value, for the copper vicinal surfaces (11α) with α = 3, 5, 7 (**left** panel) and low-index surfaces (**right** panel), as measured by Lapujoulade et al. [43,44] with a He beam incident momentum *k_i_* = 6.35 Å^−1^ (*k_i_* = 11 Å^−1^ for Cu(111)) and incident angles *θ_i_* indicated in the panels. The arrows indicate the roughening transition temperature *T_R_* [43]; the slight slope increase for Cu(001) above 460 K can hardly be distinguished from the effect of anharmonicity [43].

A selection of HAS specular reflectivity data measured by Lapujoulade et al. [43,44] as a function of the surface temperature for the vicinal surfaces of copper (11α), with α = 3, 5, 7, is reproduced in Figure 2 (left panel) and compared with the data for the low-index surfaces (110), (111) and (001) (α = 0, 1, ∞) (right panel). The reflectivity data, normalized to the extrapolated zero-temperature value *I*(*k_i_*, 0), were plotted on a logarithmic scale so as to give the temperature dependence of the DW exponent, with the corresponding He beam incident wave vectors *k_i_* and angles *θ_i_* indicated in the panels. The HAS experimental points were fitted by Lapujoulade et al. (full lines) [43,44] with a theory for the DW temperature dependence based on a He-atom surface phenomenological potential. The surface dynamics were described by a surface Debye temperature of the order of 230 K and additional adjustable parameters to account for the Beeby correction, for the so-called Armand effect, and for anharmonic corrections. The latter correction accounts for high-temperature deviations from the expected linearity.

The fittings in Figure 2 are excellent for the densely packed surfaces (111) and (001), which showed a regular behavior up to the highest measured temperature of 800 K. The theory appeared to be insufficient to explain the sudden decrease in intensity observed above the temperature *T_R_* for Cu(113), Cu(115), Cu(117) and Cu(110). This led Lapujoulade et al. [8,9] to consider the sudden decrease in intensity as clear evidence for a roughening transition, promoted by and affecting first the step rows. For the first three surfaces, the roughening temperature was of the order of room temperature or larger for Cu(110). Figure 2 shows evidence of roughening for the closed-packed surface Cu(111).

Table 1 lists the values of *λ*_HAS_ calculated with Equation (2) from the data in Figure 2. The values of *λ*_HAS_ below and above the roughening transition are shown in lightface and boldface, respectively. In Equation (2), the work function *ϕ* = 4.53 eV measured by Gartland et al. [52] for the Cu(112) surface was adopted for the other vicinal surfaces. This was, however, very close to that of the Cu(001) surface, which actually was the terrace plane for α = 3, 5, 7. The Cu(001) terrace values were then used also for *n_s_*, and *a_c_.*

At temperatures below the roughening transition, the intensity slope used in Equation (2) was that of the interpolating line calculated by Lapujoulade et al. from the data shown in Figure 2 [31] and including anharmonic corrections, while above the transition, the experimental intensity ratio at the two temperatures *T*_1_ and *T*_2_ reported in Table 1 was used. For the Cu(111) surface, two different sets of data from Ref. [31] are reproduced in Figure 2, corresponding to the different incident angles. Although the slopes were somewhat different, the corresponding values of *λ*_HAS_ reported in Table 1 were about the same, as expected. For the Cu(001) surface, two different values of *λ*_HAS_ were associated with the slight decrease in the intensity observed with respect to the fitting curve, although this small effect could be hardly associated with a real transition rather than with a gradual increase in surface disorder.

An interesting result from this analysis was the appreciable increase in the electron–phonon interaction above the roughening transition. As anticipated above, this appeared to be consistent with what was recently reported for a CdTe surface [48], although the mechanism for the electron–phonon interaction at a roughened metal surface may be rather different from that at an intrinsic semiconductor surface. It should be noted that disorder activates additional scattering channels at the expense of ordinary specular and diffraction channels. Thus, a steeper decay of the DW exponent observed at increasing temperature above the disorder threshold associated with roughening could be, in part, an effect of opening new competitive scattering channels, rather than an indication of a larger electron–phonon interaction. On the other hand, below the disorder threshold, the observed increase in *λ*_HAS_ had a solid basis, relying on the localization of both electronic and phonon excitations, conferring a quasi-one-dimensional character to step dynamics. Phonon softening at the surface steps [57] may be viewed as a manifestation of a larger local electron–phonon interaction.

**Figure 3:**
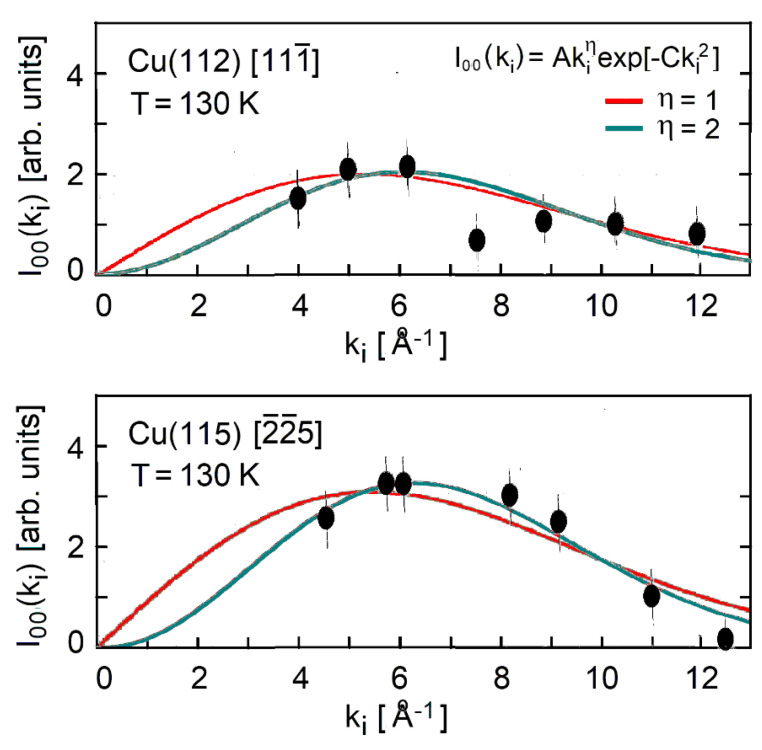
Specular HAS intensity as a function of the incident wave vector for the copper vicinal surfaces Cu(112) and Cu(115), measured at a surface temperature of 130 K along the directions normal to the steps $\left[11\bar{1}\right]$ and $\left[22\bar{5}\right]$, respectively, (●, from Miret-Artés et al. [46]). Solid lines show the fits of the HAS data with Equation (6) (reproduced in the upper panel) with two different values of the exponent *η*.

The HAS reflectivity was measured by Miret-Artés et al. [46] for the Cu(112) and Cu(115) vicinal surfaces, along the $\left[11\bar{1}\right]$ and $\left[\bar{5}\bar{5}2\right]$ directions, respectively. In Figure 3, their specular intensities from the vicinal plane (*θ_i_* = 45°) are plotted for seven different values of the incident *k_i_* at the same temperature of about 130 K. The maximum value of $k_{iz,\max}^{2}$ was about 15 Å^−2^ for both surfaces [46]. For both surfaces, the specular intensity was fitted with the function

(6) $$I_{00}(k_{i})=Ak_{i}^{\eta }\exp[-Ck_{i}^{2}],$$

where the exponential represents the DW factor, and *A* and *C* are two fitting constants. For the (112) surface, the dip in intensity at *k_i_* = 7.5 Å^−1^ was presumably due to a bound-state resonance and did not allow to clearly distinguish between the fits with the two values of *η* and the respective values of *k_i,_*_max_. The fittings in Figure 3 for the Cu(115) surface suggested *η* = 2 with a maximum at $k_{i,\max}^{}≅\;\;7.0$ Å^−1^ rather than *η* = 1 with $k_{i,\max}^{}≅\;\;5.5$ Å^−1^.

Table 2 lists the corresponding values of *λ*_HAS_ at *T* = 130 K derived from the dependence of the specular HAS intensity on the incident wave vector (Equations (3,4)) for the surfaces Cu(112) and Cu(115) and Al(221) and Al(332). A comparison of the results for *η* = 1 and 2 clearly showed that the values of *λ*_HAS_ increased with *η.* The resulting values of *λ*_HAS_ for Cu(115), even with *η* = 1, turned out to be systematically larger than those obtained from the temperature dependence of the DW exponent (Equation (3)) in Table 1 in the low-temperature region). They were more consistent with the values found above the roughening transition temperature in Table 1. For Al(221) and Al(332), the results in Table 1 and Table 2 are similar.

It should be noted that the terraces of the Cu(112) surfaces are (111) surfaces, while the terraces of the other Cu(11α) surfaces (α = 3, 5, 7) are (001) planes. For vicinal surfaces with a short inter-step period and a densely packed terraces like Cu(112), the actual crystallographic unit cell area (*a_c_* = 15.9 Å^2^) should be a better choice than the value of the corresponding terrace surface (*a_c_* = 5.64 Å^2^). Table 2 shows that with the larger value of *a_c_*, *λ*_HAS_ dropped to 0.10 for *η* = 1 (close to that of Cu(001) and Cu(111)) and to 0.16 for *η* = 2 (Table 2).

It appeared, however, as a general fact, that *λ*_HAS_ increased when moving from high-index to intermediate vicinal surfaces. The largest *λ*_HAS_ of the Cu(11α) series derived from the DW temperature dependence was found for α = 5. Such an increase in the surface el–ph interaction can be associated with the presence of steps, as long as they were sufficiently localized but not too far apart. The localization of the surface electronic states at the Fermi level could also stay behind the increase in *λ*_HAS_ above the roughening transition.

## 4. The Aluminum Vicinal Surfaces Al(221) and Al(332)

**Figure 4:**
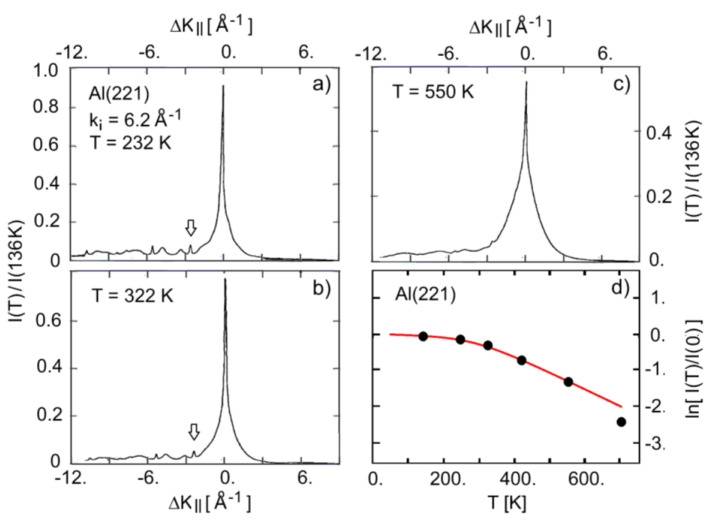
(**a**–**c**) HAS angular distributions from the Al(221) surface around the specular peak at three different surface temperatures and the same incident wave vector *k_i_* = 6.2 Å^−1^ as functions of the parallel wave vector change in the direction $\left[\bar{1}\bar{1}4\right]$ normal to the steps [54]. Intensities are shown in units of the specular intensity at *T* = 136 K. The arrows in panels (**a**,**b**) indicate the positions of possible features from down-hill terrace specular scattering. (**d**) Temperature dependence of the DW exponent. The linear part of the fit (red full line above 300 K) indicates *λ*_HAS_ = 0.71, while the point at 712 K would be fitted by a slope in the 550–712 K range corresponding to *λ*_HAS_ = 1.33.

The scattered HAS intensity at a total angle of $\theta_{i}+\theta_{f}$ = 91.5° was measured for the vicinal surfaces Al(221) [47,54,55] and Al(332) [47,48,55], as a function of both the surface temperature and the incident wave vector. Figure 4a–c reproduces some of the HAS angular distributions from the Al(221) surface measured by Witte et al. [54,55] at three different surface temperatures and given an incident wave vector *k_i_* = 6.2 Å^−1^. They were plotted as functions of the parallel wave vector change ΔK_||_ in the direction $\left[\bar{1}\bar{1}4\right]$ normal to the steps. Specular scattering from the crystallographic surface occurred at ΔK_||_ = 0, while specular scattering from the terraces occurred at about ±2.4 Å^−1^, the sign corresponding to either the up-hill or down-hill scattering configuration. Panel (d) shows the DW exponent derived from the full set of data points reported by Witte et al. and a $\coth(\theta_{D}/2T)$ fit (red full line), where *θ_D_* = 790 K is the aluminum Debye temperature [58]. The corresponding *λ*_HAS_ from Equation (2) and the parameters listed in Table 1, when referred to the approximate linear behavior in the temperature interval 232–550 K, was 0.71. The experimental point at 712 K clearly deviated from the fitting curve, with the larger DW slope in the high-*T* range giving *λ*_HAS_ = 1.33. Such a large increase above 550 K could be interpreted as an effect of surface roughening, similar to what observed for Cu(11α). Clearly, more measurements in this range should be made available in order to confirm this interpretation.

**Figure 5:**
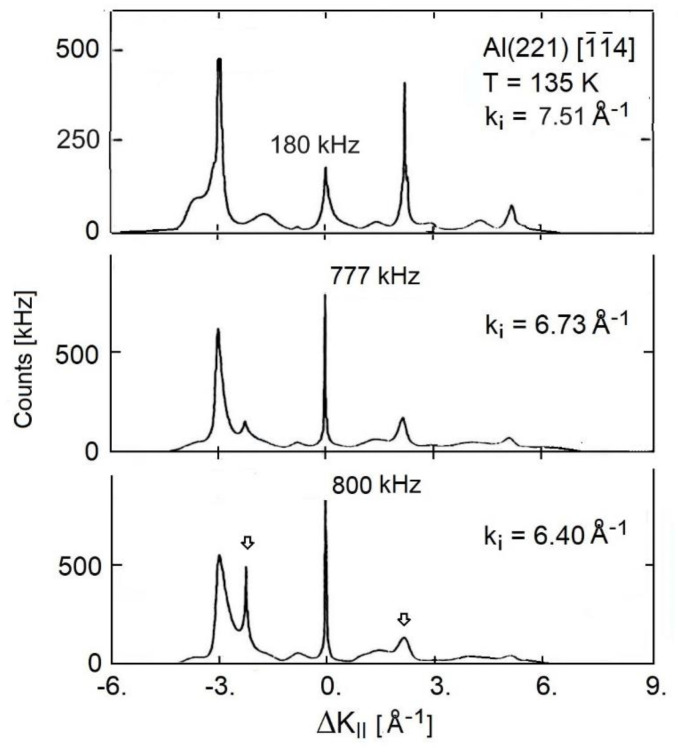
HAS angular distributions from the Al(221) surface at the surface temperature of 135 K for three different incident wave vectors *k_i_* as functions of the parallel wave vector change ΔK_||_ in the direction $\left[\bar{1}\bar{1}4\right]$ normal to the steps (adapted from [48]). The exponential drop of the specular intensity (ΔK_||_ = 0) at larger *k_i_* (see Equation (6)) is evident at *k_i_* = 7.51 Å^−1^ (top panel). The maximum specular intensity was found at *k_i,_*_max_ ≅ 6.5 Å^−1^. The arrows in the lowest panel indicate the positions of possible features from either up-hill (positive ΔK_||_) or down-hill (negative ΔK_||_) terrace specular scattering.

The value of *λ*_HAS_ found for Al(221) below 550 K was, however, much larger than the one found for the Al(111) surface (*λ*_HAS_ = 0.30, see Table 2 of Ref. [31]), as well as the bulk value *λ* = 0.43 ± 0.05 [59], but it almost coincided with the value of *λ*_HAS_ = 0.72 derived via Equations (3,4) from the wave vector dependence of the specular HAS intensity at *T* = 135 K and for *η* = 1 (Figure 5). It may be argued that also for the vicinal surface Al(221), a large increase in the el–ph interaction occurred with respect to that at the low-index surfaces, with a further increase above 550 K as a possible effect of roughening. Note that in Al(221), the specular scattering from the crystallographic (221) surface was still larger than or comparable to that from the (111) terraces (small arrows in Figure 4 and Figure 5), despite the fairly large inter-step distance of 8.74 Å.

**Figure 6:**
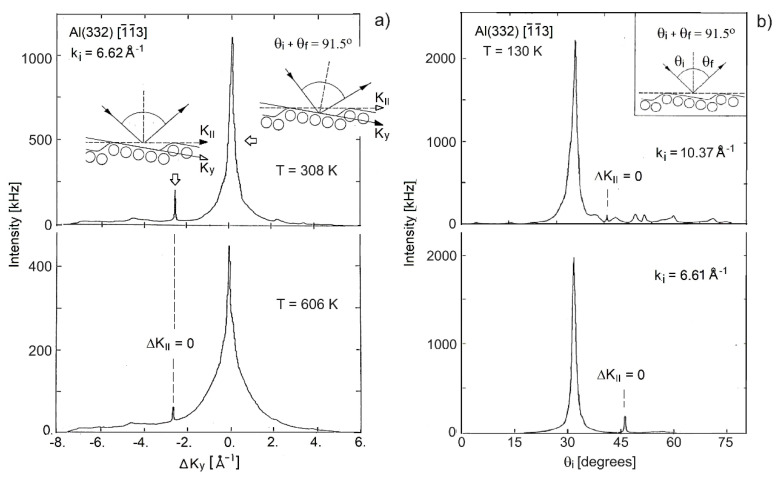
HAS angular distributions from Al(332) along the direction $\left[\bar{1}\bar{1}3\right]$ normal to the steps in the up-hill orientation (adapted from Lock et al. [47,48]). (**a**) The results at two different surface temperatures (302 K and 605 K) and a given incident wave vector *k_i_* = 6.62 Å^−1^. (**b**) The results for different incident wave vectors (*k_i_* = 10.37 and 6.61 Å^−1^) at a given surface temperature of 130 K. The insets in (**a**) show the up-hill scattering configuration, either specular with respect to the (332) surface (left side) and yielding a small elastic peak, or specular with respect to the (111) terraces (right side), and yielding a strong elastic peak. The inset in (**b**) illustrates the up-hill scattering configuration from the vicinal surface (113) with a total scattering angle of 91.5°. The large peaks were derived from terrace scattering, while the scattering from the (332) surface (ΔK_||_ = 0) yielded very small peaks.

Figure 6, adapted from Lock et al. [48,49], shows the angular distributions from the vicinal surface Al(332). Figure 6a shows the changes of the HAS specular intensities from both terraces and step arrays observed by changing the temperature at a given *k_i_*, while Figure 6b displays the intensity when changing *k_i_* at a given temperature. This surface with its large 14.30 Å inter-step distance (see Figure 1), exhibited, instead, a dominant HAS specular scattering from the (111) terraces ($\Delta K_{y}=0$) and only small sharp peaks from the periodic array of the steps of the (332) surface (ΔK_||_ = 0). The small peaks at ΔK_||_ = 0 in Figure 6a evaluated by Equation (2), with the parameters listed in Table 1 and in the temperature interval of 308–606 K led to the value of *λ*_HAS_ = 0.61. The same value was obtained from the wavelength dependence in the *k_i_* interval of 6.61–10.37 Å^−1^ (Figure 6b) from Equations (3)–(5), with *η* = 1. The same consistency was found for Al(221), which strongly supported the choice of *η* = 1.

It was noted that *λ*_HAS_ for Al(332), although still larger than that of Al(111), turns out to be smaller than that found for Al(221). An interesting question is whether this was due to the different sources of data used for Al(332) (specular HAS from the (111) terraces) and Al(221) (specular HAS from the periodic array of steps of the (221) surface). The temperature and incident wave vector dependence of the small peaks at ΔK_||_ = 0 in Figure 6 permitted to compare terraces (see Figure 1) and steps at $\Delta K_{y}=0$ and to assess their respective contributions to the surface electron–phonon coupling. The second row of Al(332) data in Table 1 shows indeed that *λ*_HAS_ derived from the temperature dependence of the small ΔK_||_ = 0 peak increased to 1.10, which brought the surface electron–phonon coupling of Al(223) above that of Al(221) and much above that of the Al(111) low-index surface. A similar increase could probably be determined from the *k_i_* dependence of the ΔK_||_ = 0 peak, although the very small peak at the higher wave vector was hidden in the background.

**Figure 7:**
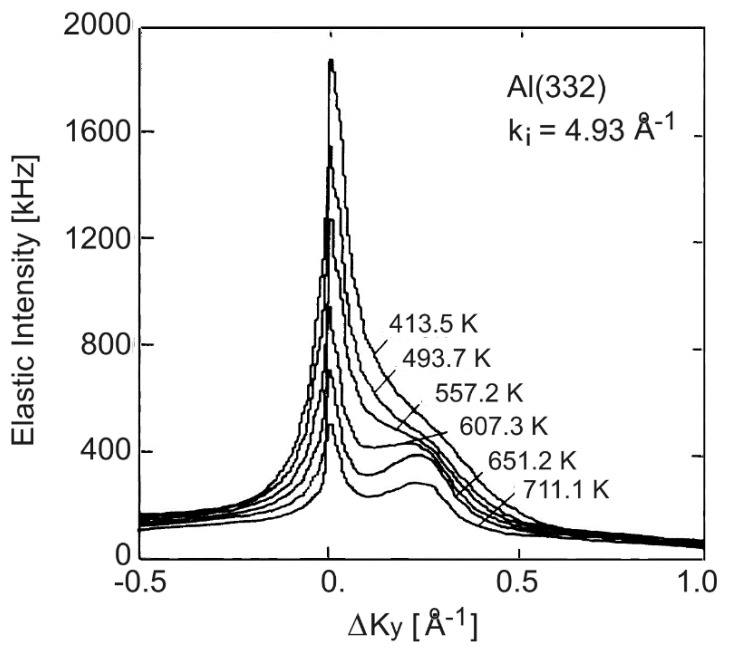
HAS angular distributions measured at around $\Delta K_{y}=0$ from the (111) terraces of Al(332) at different temperatures and with a fixed incident wave vector *k_i_* = 4.93 Å^−1^. With increasing temperature, an additional peak appeared which was attributed to faceting or increasing disorder (adapted from Lock et al. [47,48]).

Figure 7, adapted from Lock et al. [47,48], illustrates a problem inherent in determining el–ph mass enhancement factors from stepped surfaces. The temperature dependence of the angular distributions from Al(223) terraces in Figure 7 could hardly be used to extract *λ*_HAS_ due to the partial superposition of an additional peak growing with increasing temperature at positive values of $\Delta K_{y}$. This was attributed to a temperature-driven instability towards faceting at the steps. Nevertheless, the value of *λ*_HAS_ = 0.66 obtained from Equation (2) in the lowest temperature interval (413.5–493.7 K) (see Table 1, third row for Al(332)), where the specular scattering intensity from the (111) terraces was still dominant, was consistent with the terrace values derived from Figure 6. At higher temperatures, where faceting instability occurs, it can make sense to consider the areas of the double peak features and to determine the logarithm of the ratio of two areas measured at two different temperatures in order to obtain overall qualitative information on the electron–phonon interaction. The fourth entry under Al(332) in Table 1 shows a comparatively large value of *λ*_HAS_ = 1.42 obtained in this way over the entire 414–711 K temperature range. This is consistent with *λ*_HAS_ = 1.33 found at similar high temperatures for Al(221). The large values of *λ*_HAS_ were attributed to a roughening transition, in analogy to what was reported for copper vicinal surfaces.

It is worth noting that the enhancement of *λ*_HAS_ at vicinal surfaces with respect to that of the terrace high-index surface was appreciably stronger for aluminum than for copper. This can be attributed to the rather different range of the surface el–ph interaction for the two metals, expressed by *n_s_*, which was rather short for the aluminum surfaces and quite longer for the copper surfaces. As discussed in Ref. [31], this difference is in turn related to the rather different values of the surface Fermi wave vectors at the nesting points associated with the el–ph interaction. The dependence of this interaction on surface morphology is naturally more pronounced the shorter is its extension into the bulk.

## 5. Conclusions

The method of extracting the electron–phonon interaction (mass enhancement factor *λ*_HAS_) at conducting surfaces from the Debye–Waller exponent of helium atom scattering [31] was applied to the analysis of existing HAS data on Cu and Al vicinal surfaces. It was found that the electron–phonon interaction of the vicinal surfaces analyzed in the present work was generally larger than that of the corresponding low-index surface of the terraces between two neighboring steps. The observed increase in *λ*_HAS_ was attributed to the specific contribution of the steps. The case of Al(332) was illuminating in this respect, because it permitted to compare the value of *λ*_HAS_ obtained from the HAS reflectivity of the (111) terraces to that obtained from the HAS reflectivity of the crystallographic surface (332).

More intriguing is the pronounced increase in *λ*_HAS_ at higher temperature where some form of disorder appeared, such as roughening or terrace faceting. Figure 7 shows that disorder activated additional scattering channels, which subtracted intensity from specular and diffraction scattering, leading to a steeper decay of the DW exponent with the temperature. It is expected that disorder induced by increasing temperature starts from steps were atoms are more loosely bound at their lattice positions. On the other hand, the larger contributions to *λ*_HAS_ of the step atoms compared to the terrace atoms, as found in the present analysis, was likely to be due to the step localization of both electronic and phonon excitations, which acquired a quasi-one-dimensional character, so that phonon softening at the steps [57] might be linked to the larger local electron–phonon interaction.

The present work was stimulated by existing HAS measurements which were not intended for the derivation of *λ*_HAS_, the method being unknown at the time of the measurements. There are very interesting HAS investigations also on clean and Fe-covered Pt(997) surfaces [24], where unfortunately the missing normalization of the HAS diffraction spectra measured at several different temperatures does not permit the evaluation of *λ*_HAS_. We hope that this study will stimulate new HAS studies explicitly designed for the derivation of both *λ*_HAS_ and the mode-selected *λ_Qν_*. The recent studies on the possible superconductivity enhancement as an effect of surface disorder [60] offer further good reasons for new HAS studies specifically aiming at the derivation of the electron–phonon interaction at stepped surfaces and conducting surfaces with various types of disorder.

## Figures and Tables

**Table 1:** Electron–phonon mass enhancement factor HAS for the Cu(11) surfaces (Figure 2) and for the Al(221) and Al(332) vicinal surfaces, derived from the dependence of the specular HAS intensity on the surface temperature (Equation (2)). The results are listed both for ordered (low-temperature *T*_1_–*T*_2_ interval) and for roughened (high-temperature *T*_1_–*T*_2_ interval, boldface entries) vicinal surfaces. No roughening transition was observed for Cu(111). In contrast to the large values for Al(221) and Al(332), the mass enhancement factor reported for Al(111) was *λ*_HAS_ = 0.30 [31].

Surface	*ϕ*	*n_s_*	*a_c_*	$k_{iz}^{2}$	*D*	*T*_1_–*T*_2_	$\ln\frac{I(T_{1})}{I(T_{2})}$	*λ*_HAS_
	[eV]		[Å^2^]	[Å^−2^]	[meV]	[°K]		
Cu(110) ^a^	4.48 ^b^	6.8 ^d^	9.22	35.60 ^e^	6.27 ^h^	200–500	1.20	0.11
						500–800	1.96	**0.18**
Cu(111) ^a^	4.94 ^b^	8.5 ^d^	5.64	20.34 ^e^	8.85 ^h^	500–800	0.70	0.12
				36.09 ^f^		500–800	1.09	0.13
Cu(113) ^a^	4.53 ^c^	6.8 ^d^	6.52 ^n^	35.60 ^e^	6.35 ^h^	200–500	1.04	0.13
						500–800	2.20	**0.28**
Cu(115) ^a^	4.53 ^c^	6.8 ^d^	6.52 ^n^	13.27 ^e^	6.35 ^h^	200–500	0.74	0.18
						500–800	1.37	**0.33**
Cu(117) ^a^	4.53 ^c^	6.8 ^d^	6.52 ^n^	13.27 ^g^	6.35 ^h^	100–400	0.47	0.11
						400–700	0.84	**0.20**
Cu(001) ^a^	4.59 ^b^	6.8 ^d^	6.52	12.94 ^a^	9.70 ^o^	200–500	0.49	0.10
						500–800	0.69	**0.14**
Al(221) ^i^	4.26 ^k^	1.6 ^d^	7.09 ^n^	21.91 ^k^	7.0 ^h,l^	232–550	1.17	0.71
						550–711	1.12	**1.33**
Al(332) ^j^	4.26 ^k^	1.6 ^d^	7.09 ^m,n^	20.70 ^t^	7.0 ^h^	308–606	0.92	0.61
				21.34 ^s^	7.0 ^h^	308–606	1.68	1.10
				11.83		414–494	0.20	0.66
						414–711	1.59	**1.42**

^(a)^ Refs. [41,42]. ^(b)^ Ref. [52]. ^(c)^ The value measured for Cu(112) [52] was adopted for all present vicinal surfaces. ^(d)^ Value for the corresponding terrace surface from Ref. [31]. ^(e)^ Ref. [43]; for Cu(111) from the data in Figure 2 with *θ_I_* = 65.8°. ^(f)^ For Cu(111) from the data in Figure 2 with *θ_I_* =56.9°. ^(g)^ Ref. [44]. ^(h)^ Ref. [51]. ^(i)^ Refs. [47,53,54,55]. ^(j)^ Refs. [47,48,55]. ^(k)^ Ref. [53]. ^(l)^ Potential depth for the (001) surface; the one for the terrace (111) surface was not available. ^(m)^ Diffuse elastic intensity (see Figure 13 of Ref. [48]). ^(n)^ Unit cell area of the corresponding terrace surface. ^(o)^ Ref. [56]. ^(t)^ Specular scattering from the terrace surface (Figure 6a [47,48]). ^(s)^ Specular scattering from the (332) surface (Figure 6a [47,48]).

**Table 2:** Electron–phonon mass enhancement factor *λ*_HAS_ derived from the dependence of the specular HAS intensity on the incident wave vector (Equations (3) and (4)).

Surface	*ϕ*	*n_s_*	*a_c_*	*k_i,_*_max_	*D*	*T*	*η*	*λ*_HAS_
	[eV]		[Å^2^]	[Å^−2^]	[meV]	[°K]		
Cu(112) ^a^	4.53 ^b^	6.8 ^c^	5.64 ^d^	~5.0	8.25 ^e^	130	1	0.29
				~6.5			2	0.45
			15.9 ^i^	~5.0	8.25 ^e^	130	1	0.10
				~6.5			2	0.16
Cu(115) ^a^	4.53 ^b^	6.8 ^c^	6.52 ^d^	~5.5	6.35 ^e^	130	1	0.26
				~7.0			2	0.38
Al(221) ^f^	4.26 ^h^	1.6 ^c^	7.09 ^d^	~6.5	7.0 ^e^	135	1	0.72
Al(332) ^g^	4.26 ^h^	1.6 ^c^	7.09 ^d^	~7.2	7.0 ^e^	130	1	0.61

^(a)^ Ref. [46]. ^(b)^ Ref. [52]; the value measured for Cu(112) was also used for Cu(115). ^(c)^ Value for the corresponding terrace surface from Ref. [31]. ^(d)^ Unit cell area of the corresponding terrace surface. ^(i)^ Cu(112) unit cell area. ^(e)^ Ref. [51]. The (111) terrace value was used for Cu(112). ^(f)^ Refs. [47,54,55]. ^(g)^ Refs. [47,48,55]. ^(h)^ Ref. [53].

## Data Availability

Data are calculated for this paper.
